# Peroxisome Proliferator-Activated Receptor Alpha Mediates the Beneficial Effects of Atorvastatin in Experimental Colitis

**DOI:** 10.3389/fimmu.2021.618365

**Published:** 2021-08-09

**Authors:** Paulo José Basso, Helioswilton Sales-Campos, Viviani Nardini, Murillo Duarte-Silva, Vanessa Beatriz Freitas Alves, Giuliano Bonfá, Cassiano Costa Rodrigues, Bruno Ghirotto, Javier Emílio Lazo Chica, Auro Nomizo, Cristina Ribeiro de Barros Cardoso

**Affiliations:** ^1^Departamento de Bioquímica e Imunologia, Faculdade de Medicina de Ribeirão Preto, Universidade de São Paulo, Ribeirão Preto, Brazil; ^2^Departamento de Análises Clínicas, Toxicológicas e Bromatológicas, Faculdade de Ciências Farmacêuticas de Ribeirão Preto, Universidade de São Paulo, Ribeirão Preto, Brazil; ^3^Departmento de Imunologia, Instituto de Ciências Biomédicas, Universidade de São Paulo, São Paulo, Brazil; ^4^Instituto de Ciências Biológicas e Naturais, Universidade Federal do Triângulo Mineiro, Uberaba, Brazil

**Keywords:** Inflammatory bowel diseases, ulcerative colitis, crohn’s disease, therapy, statins, dextran sodium sulfate

## Abstract

The current therapeutic options for Inflammatory Bowel Diseases (IBD) are limited. Even using common anti-inflammatory, immunosuppressive or biological therapies, many patients become unresponsive to the treatments, immunosuppressed or unable to restrain secondary infections. Statins are cholesterol-lowering drugs with non-canonical anti-inflammatory properties, whose underlying mechanisms of action still remain poorly understood. Here, we described that *in vitro* atorvastatin (ATO) treatment was not toxic to splenocytes, constrained cell proliferation and modulated IL-6 and IL-10 production in a dose-dependent manner. Mice exposed to dextran sulfate sodium (DSS) for colitis induction and treated with ATO shifted their immune response from Th17 towards Th2, improved the clinical and histological aspects of intestinal inflammation and reduced the number of circulating leukocytes. Both experimental and *in silico* analyses revealed that PPAR-α expression is reduced in experimental colitis, which was reversed by ATO treatment. While IBD patients also downregulate PPAR-α expression, the responsiveness to biological therapy relied on the restoration of PPAR-α levels. Indeed, the *in vitro* and *in vivo* effects induced by ATO treatment were abrogated in *Ppara* ^-/-^ mice or leukocytes. In conclusion, the beneficial effects of ATO in colitis are dependent on PPAR-α, which could also be a potential predictive biomarker of therapy responsiveness in IBD.

## Introduction

Statins are the most effective drugs used in lowering serum cholesterol levels, preventing or treating atherosclerosis and coronary heart disease (1, 2). They act by selectively inhibiting the 3-hydroxy-3-methylglutaryl coenzyme A (HMG-CoA) reductase, a key regulatory and rate-controlling enzyme of cholesterol biosynthesis (3).

The cholesterol-lower drugs have also been shown to exert cholesterol-independent or “pleiotropic” effects, which comprise anti-inflammatory, immunomodulatory, vasodilatory, antioxidative, antithrombotic and osteomodulatory activities (4–6). Several mechanisms have been suggested to explain the wide range of statins functions and most of them arise from the inhibition of isoprenoid intermediates produced during the mevalonate pathway (6). These lipid mediators are essential to structure and function of various cell-signaling proteins, including the super family of small GTPases (e.g., Ras, Rho, Rac) associated to cell traffic, cycle, and differentiation (7, 8). Thus, statins may be used to treat other conditions not related to hypercholesterolemia, including autoimmune and inflammatory diseases (9–11). Accordingly, a few experimental and clinical studies have used different types of statins to treat Inflammatory Bowel Diseases (IBD), a group of chronic and recurrent inflammatory disorders of the digestive tract, which includes Crohn’s Disease (CD) and Ulcerative Colitis (UC) (12).

Both CD and UC share clinical similarities, but they comprise unlike characteristics to be considered as autonomous entities, such as the distribution and types of lesions in the gastrointestinal tract, the histopathology, the dynamic of immune responses and the treatment responsiveness (13–15). HMG-CoA inhibitors in experimental models of colitis have been shown to attenuate the clinical signs of disease and improve the survival rates in mice (16–18). Studies in humans are scarce, but Grip and colleagues described that CD patients treated with atorvastatin (ATO) had a reduction in both disease activity and inflammatory markers, including plasma C-reactive protein (CRP) and soluble TNF receptor II (sTNFRII) levels (19). Although some underlying mechanisms have been identified as responsible for the benefits of statins in IBD, it is still unknown whether this drug can modulate inflammation in a cholesterol-independent manner.

It has been evidenced that statins can activate peroxisome proliferator-activated receptors (PPARs), a group of three transcription factors (PPAR-α, PPAR-β/δ and PPAR-γ) involved in inflammation and metabolism (20). PPARs translocate from cytoplasm into nucleus after binding to cognate ligands, heterodimerize with retinoid-X receptor (RXR) and then bind to PPAR-responsive elements (PPREs) to activate the transcription of several target genes (20). The use of statins was showed to improve the outcome of inflammatory conditions such as experimental autoimmune encephalomyelitis (EAE) and LPS-induced acute inflammation through PPAR-α activation (21, 22). However, it is unknown whether PPAR-α plays any role in ameliorating IBD outcome after statin therapy. Therefore, here we hypothesized that statins could improve the outcome of experimental colitis by modulating PPAR-α expression or activation, which could in turn impact the resolution of the ongoing pathogenic intestinal inflammation.

## Material and Methods

### Animal Studies

Male C57BL/6J wild type (WT) and transgenic *Ppar* ^-/-^ mice (6-8 weeks old) were bred and maintained in the animal facility at School of Pharmaceutical Sciences of Ribeirão Preto, University of São Paulo. Mice were housed in controlled (25°C ± 2°C and 12h/12h light/dark cycles) and in specific pathogen free conditions along with water and food *ad libitum.* All experimental procedures were approved according to the guidelines of the Ethics Committee of the University of São Paulo - *campus* Ribeirão Preto (protocol number 10.1.499.53.8) and performed according to the criteria outlined by the Brazilian Society for Laboratory Animal Science (SBCAL).

### Acute Colitis Induction and Experimental Protocol

Mice were randomly assigned to one of the three groups: untreated healthy animals, mice exposed to 3% (w/v) DSS (molecular weight: 36,000–50,000, MP Biomedicals, Illkirch, France) in drinking water and treated with vehicle (normal saline) or mice exposed to 3% DSS and treated with ATO (10mg/kg/day, Lipitor^®^, Pfizer, SP, Brazil). Healthy mice treated with ATO were not depicted since they did not show any difference compared to the untreated healthy group. DSS was provided *ad libitum* for 6 consecutive days in drinking water. ATO was suspended in normal saline and given orally to mice for 3 consecutive days from day 3 after the onset of colitis induction, once a day, until the day before euthanasia. Mice were euthanized on the 6^th^ day after colitis induction and had the blood, intestine (colon), spleen and mesenteric lymph nodes (MLN) collected for further analysis. Colon samples were divided into smaller fragments of approximately 1 cm. Then, one of these fragments was immersed in PBS/10% formaldehyde for paraffin embedding and histopathological analysis (distal colon). The other fragments were immediately frozen in liquid nitrogen for total RNA extraction (middle colon) or for measurements of cytokines levels (distal colon) or eosinophil peroxidase (EPO; middle colon), myeloperoxidase (MPO; proximal colon) and N-acetylglucosaminidase (NAG; proximal colon) activities.

### Clinical, Overall, Postmortem and Accumulated Scores

Clinical signs and overall, post-mortem or accumulated scores were performed as previously described (23). Briefly, clinical parameters such as daily weight loss (≥5% and <10%), wet anus, diarrhea, bleeding stools, hypoactivity and piloerection were evaluated and received 1 point each. Weight reduction of ≥10% compared to previous day received 2 points. The overall score was resulted of the sum of all daily clinical scores per mouse. Following the same scoring criteria used to clinical signs analysis, the post-mortem score was performed by evaluating local or diffuse diarrhea/bleeding in the colon. Local alterations received 1 point, while diffuse changes and stenosis received 2 points. The accumulated score was defined as sum of clinical score at the day of euthanasia and post-mortem score, as previously described (23).

### Total Leukocytes and Differential Cell Counting

Total circulating leukocytes were counted using a Neubauer chamber and blood smears processed for Romanowsky staining (Laborclin Products for Laboratory Ltda, Pinhais, PR, Brazil) for differential cell counting.

### Histology and Histopathological Analyzes

The colon fragments previously immersed in PBS/10% formalin were processed for paraffin embedding followed by microtomy of 5 μm sections, which were stained with hematoxylin and eosin. The histopathological analysis was based on a previous study with some modification (24). Briefly, mucosa thickness, lamina propria (LP) cellularity, inflammatory infiltrate and epithelial morphology were evaluated. The images were captured using both 20x and 40x objectives through a digital video camera (Evolution MP 5.0-color, Media Cybernetic, Silver Spring, MD, USA) coupled to a light microscope (Nikon, Eclipse 50i, Melville, NY, USA). An area of 1,57 mm^2^ from each colon sample was evaluated and morphometry was performed using the software “Image-Pro Insight” (Media Cybernetics, Rochville, MD, USA). The cells in the LP were counted and the results were expressed as number of cells per mm^2^.

### Cytokines Quantification by ELISA

The cytokines were quantified by ELISA on cell culture supernatants and/or colon tissue lysate (IL-4, IL-6 and IL-10 - BD Biosciences, San Jose, CA, USA; IL-17A – Abcam, Cambridge, MA, USA) according to the manufacturer’s instructions.

### Myeloperoxidase (MPO), Eosinophil Peroxidase (EPO) and N-Acetylglucosaminidase (NAG) Activities Measurement

MPO, NAG and EPO activities were measured as described previously (25). For MPO assay, small colonic fragments were homogenized in buffer containing 0.1 M NaCl, 0.02 M NaPO_4_, and 1.015 M NaEDTA, pH 4.7 and resuspended in a hypotonic solution to erythrocytes rupture followed by three freeze-thaw cycles to lyse the remaining cells. After centrifugation, the supernatant was incubated with 3,3’,5,5’-tetramethylbenzidine (TMB Substrate Reagent Set – BD OptEIATM, San Diego, CA) at 37°C for 15 minutes, the reaction stopped using 2 N H_2_SO_4_ and the optical density (OD) measured at 450 nm. For NAG activity, the same supernatant from MPO assay was used and then incubated with 2.24 mM p-nitrophenyl-2-acetamide-β-D-glucopyranoside and 50 mM citrate buffer (pH 4.5) for 60 minutes at 37°C. The reaction was stopped using 0,2 M glycine buffer (pH 10.4) and absorbance was determined at 405 nm. For EPO measurement, colonic fragments were homogenized in HBSS buffer and then subjected to both hypotonic lysis and freeze–thaw cycles. The supernatant obtained by centrifugation was incubated with o-phenylenediamine dihydrochloride (SIGMAFAST OPD tablet set, Sigma-Aldrich, Saint-Louis, MO, USA) for 30 min at 37°C. The reaction was stopped using 2 N H_2_SO_4_ and absorbance was determined at 492 nm. All the results were expressed as optical density per gram of tissue.

### RNA Extraction, Reverse Transcription, and Quantitative Real-Time PCR (qRT-PCR)

The colonic tissues were homogenized in TRIzol Reagent (Invitrogen, Carlsbad, CA, USA) for total extraction and the mRNA was isolated using *SV Total RNA Isolation System Kit* (Promega, Madison, WI, EUA), according to manufacturer’s instructions. RNA purity and quantity were assessed by using a NanoDrop 1000 (Thermo Fisher Scientific, Wilmington, DE, EUA). The purified RNA was used to synthetize the complementary DNA with GoScript™ Reverse Transcription System (Promega, Madison, WI, USA) according to the manufacturer´s instructions. Finally, all cDNA samples were diluted in nuclease-free water. Quantitative real-time PCR (qRT-PCR) studies were performed using *GoTaq* ^®^ *qPCR Master Mix* (Promega, Madison, WI, EUA) in a StepOnePlus™ Real-Time PCR System (Applied Biosystem, Foster City, CA, EUA). cDNA sample were mixed with solution containing forward and reverse target primers, *GoTaq* ^®^ *qPCR Master Mix* and nuclease-free water as provided by the manufacturer in 96-well MicroAmp Fast Optical reaction plates (Applied Biosystem, Foster City, CA, EUA). Gene-specific primers for mouse IL-4, IL-17A, PPAR-α and β-actin were used and their respective sequences were as follows: IL-4 - (sense) 5’-AAG AGC ATC ATG CAA ATG GA-3’, (antisense) 5’-TTA AAG CAT GGT GGC TCA GTA C-3’; IL-17A - (sense) 5’-TGC CCT CCA CAA TGA AAA GA-3’, (antisense) 5’-AAC ACG AAG CAG TTT GGG AC-3’; PPAR-α - (sense) 5’- TCA ATG CCT TAG AAC TGG ATG A-3’, (antisense) 5’- CCGATCTCCACAGCAAATTATA-3’;

β-actin - (sense) 5’-AAC GAG CGG TTC CGA TG-3’; (antisense) 5’-GGA TTC CAT ACC CAA GAA GGA-3’. All primers were obtained from Sigma-Aldrich (Saint-Louis, MO, USA). The PCR conditions were 50°C for 2 min, 95°C for 2 minutes, 40 cycles for 15 s at 95°C, 30 s at 58°C and 30 s at 72°C, followed by melting curve determination. All the qPCR assays included a negative control (without cDNA template), and the samples were run in duplicate. β-actin was used to normalize the expression levels of target genes and the results were evaluated based on the Ct value. The differential expression of mRNA was defined by the formula 2^-ΔΔCt^, according to previous studies (26).

### Isolation of Leukocytes From Spleen and Mesenteric Lymph Nodes (MLN)

Spleen and MLNs were removed, crushed through a 70 μm cell strainer (BD Biosciences, Heidelberg, Germany) using a syringe plunger and then suspended in complete RPMI 1640 medium - supplemented with 3.7 g/L sodium bicarbonate, 5% heat inactivated fetal bovine serum (FBS; Gibco – Invitrogen, Ontario, Canada), 2 mM L-glutamine (Sigma-Aldrich, Steinheim, Germany), 1 mM sodium pyruvate (Sigma-Aldrich, Saint Louis, MO, USA), 0.5 mM 2-mercaptothanol (Sigma-Aldrich, Saint Louis, MO, USA), 10 mM HEPES (Sigma-Aldrich, Saint Louis, MO, USA), 100 IU/mL penicillin (Gibco-Invitrogen, Ontario, Canada) and 100 μg/mL streptomycin (Gibco – Invitrogen, ONT, CA) - for subsequent cell culture and flow cytometry assays. The spleen cells were subjected to additional step to lyse red blood cells with appropriate lysis buffer. Trypan blue dye (0.2%) exclusion tests were used to determine the number of live cells before cell culture and immunophenotyping experiments.

### Flow Cytometry

For phenotypic characterization of the leukocyte’s populations from spleen and MLN, monoclonal antibodies conjugated to fluorochromes were used. In brief, cells were incubated with antibodies (1x10^6^ cells/mL) for 20 minutes at 4°C in the dark. The cells were then fixed with PBS containing 1% formaldehyde or followed by intracellular staining. All antibodies (CD3, CD4, CD8, CD11b, CD11c, CD49b, IL-17, IFN-γ) were obtained from BD Pharmigen™ (San Diego, CA, USA). Leukocytes were acquired on Canto II Flow Cytometer (BD Biosciences, San Jose, CA) and the data were analyzed using FlowJo version 6.6.3 software (TreeStar, San Carlos, CA).

### Lymphocyte Proliferation and Cell Death Assays

For lymphocyte proliferation assay, the spleen cells were stained with 5 µM carboxyfluorescein diacetate succinimidyl ester (CFSE; Molecular Probes, Invitrogen, Carlsbad, USA) for 10 minutes at 37°C. Subsequently, cells (2.5x10^6^/mL) were stimulated with concanavalin A (ConA, 3 μg/mL) and incubated at 37°C/5%CO_2_ for 72 hours. Then, the supernatant was collected, and the cells analyzed by flow cytometry as described earlier. The results were expressed as the total number of divisions divided by the number of cells that went into division and termed as proliferation index.

Cell death (apoptosis and necrosis) was assessed using propidium iodide and annexin-fluorescein, according to manufacturer’s instructions (Annexin-V-FLUOS Staining kit - Roche, Mannhelm, Germany). Cells were acquired in flow cytometer and results interpreted as follows: double-positive cells (annexin^+^PI^+^) were considered as late apoptotic cells or necrotic cells; annexin^+^PI^-^ cells were considered as early apoptotic cells; annexin^-^PI^+^ cells were considered as necrotic cells; double-negative cells (annexin^-^PI^-^) were considered as live cells. Saponin (100mM) was used as positive control of cell death.

### Bioinformatics Analysis

We manually curated the Gene Expression Omnibus (GEO) repository (https://www.ncbi.nlm.nih.gov/geo/) to find transcriptome datasets of colonic regions related to “IBD (CD and UC)” in humans and “DSS-induced colitis” in mice. Expression analysis was performed based on author normalized expression values using the GEO2R software (https://www.ncbi.nlm.nih.gov/geo/geo2r/). When necessary, the normalized expression values were converted to a log_2_ scale for analysis. Probes that matched the same gene symbol were collapsed by taking the one with the lowest p-value. The heatmap was generated using the Morpheus software (https://software.broadinstitute.org/morpheus/) based on the log_2_-fold change values for each gene throughout all studies analyzed. Volcano plot was generated using limma (27). The genes depicted were significantly differentially expressed at an adjusted p-value cutoff of 0.05 (red = upregulated genes; blue = downregulated genes).

Five datasets were selected for analysis, being 4 from humans and 1 from mice. Human datasets comprised two representative expression data from UC patients and controls (GSE75214 and GSE87466) and other two from UC patients who received infliximab therapy, including responders and non-responders to drug therapy (GSE16879 and GSE12251). GSE75214 contains transcriptomic data of colonic biopsies from healthy individuals (n=11) and patients with CD (n=8) or UC (n=74). Only subjects with active disease were considered for analysis, which was confirmed by endoscopic evaluation. GSE87466 contained gene expression profiling of mucosal biopsies from controls (n=21) and patients with moderately to severely active UC (n=87). Disease activity was also endoscopically assessed. The differentially expressed genes (DEGs) in GSE75214 and GSE87466 datasets were filtered by either logFC < -1 (downregulated genes) or logFC > 1 (upregulated genes) and by adjusted p-value <0.05. Then, KEGG Pathway enrichment analysis was performed on the up and downregulated genes using the Metascape software (28). Correlation analyzes were performed between PPARA and PPARGC1A genes using data from GSE75214 and GSE87466 datasets. Based on Kolmogorov-Smirnov normality test, we selected either Pearson or Spearman analysis for the correlation analyzes.

GSE16879 contained transcriptomic data of mucosal biopsies from patients with UC before and 4-6 weeks after their first infliximab infusion. Both responders and non-responders to therapy were included in the study. Responders (n=8) and non-responders (n=16) were classified based on endoscopic and histologic findings at 4-6 weeks after first dose of infliximab treatment. GSE12251 contained transcriptomic data of UC patients 8 weeks after receiving infliximab therapy, including responders and non-responders. Responders (n=12) and non-responders (n=10) to infliximab were defined according to endoscopic and histologic mucosal healing at week 8. Mice dataset comprised one representative expression data from C57BL/6 mice subjected to 3% DSS colitis for 6 days (GSE22307). We assessed the data from normal (n=5) and sick (n=6) colonic samples.

### Data Analysis and Statistics

Statistical analysis was performed using the Graphpad Prism software. In all variables normal distribution and homogeneous variance were tested. When the distribution was considered normal, and variance was homogeneous, parametric tests were used (unpaired Student’s t test or one-way ANOVA with Tukey’s post-test). In cases of non-Gaussian distribution of data, the nonparametric tests Mann–Whitney or Kruskal–Wallis (Dunn’s) were used. Results were expressed as mean ± SEM. The observed differences were considered significant when p < 0.05 (5%).

## Results

### ATO Modulates Immune Responses *In Vitro*

**Figure 1:**
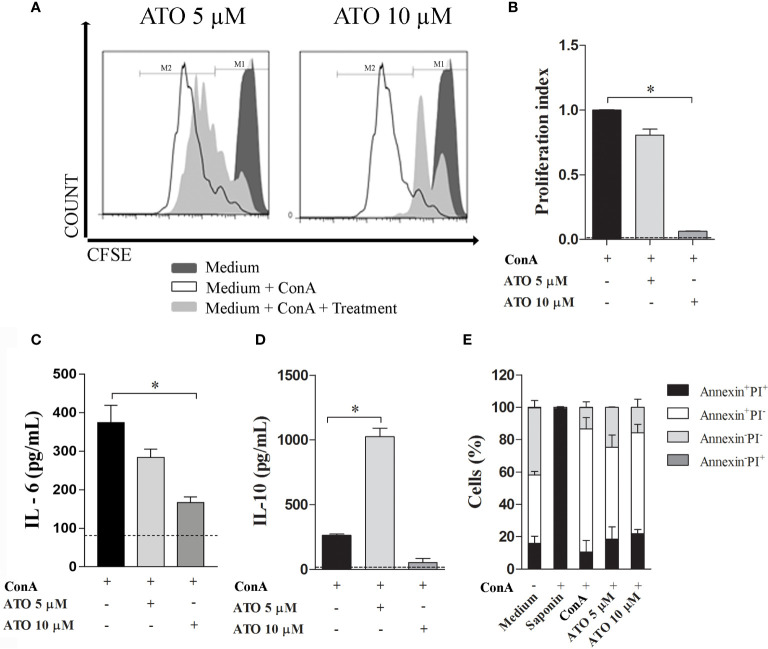
Immunomodulatory effects of ATO in *vitro*. Splenocytes from C57BL/6 WT mice were stimulated with 3 µg/mL of ConA and concomitantly treated with 5 or 10 µM ATO for 72 h. **(A)** Representative histograms of cell proliferation in CFSE-stained cells assessed by flow cytometry. **(B)** Graphic representation of proliferation index (the total number of divisions divided by the number of cells that went into division). **(C, D).** Quantification of IL-6 **(C)** and IL-10 **(D)** levels on the cell culture supernatants of ConA-stimulated cells that received or not ATO treatment. Dotted lines indicate cytokine production by unstimulated cells. **(E)** Cell death assessed by Annexin/PI staining followed by flow cytometry analysis. Double positive-staining (annexin^+^PI^+^) was considered late apoptotic or necrotic cells; annexin^+^PI^-^ was considered early apoptotic cells; annexin^-^PI^+^ were the necrotic cells; double-negative cells (annexin^-^PI^-^) were considered as live splenocytes. Saponin was used as positive control of cell death. The dashed line indicates basal values from unstimulated cells. All the results are representative of 3 independent experiments that were performed, each one, with splenocytes pooled from 3 mice. ANOVA test was used followed by Tukey comparison or Kruskal–Wallis (Dunns) test. *p < 0.05. ATO, Atorvastatin; ConA, Concanavalin A; CFSE, 5(6)-carboxyfluorescein N-hydroxysuccinimidyl ester; PI, Propidium iodide. WT, Wild type.

To evaluate the immunomodulatory properties of statins, CFSE-stained splenocytes were stimulated with ConA in the presence or absence of ATO for 72 h. Treatment with 10 μM ATO reduced the proliferation rate, whereas no difference was observed after the use of 5 μM ATO (**Figures 1A, B**, respectively). Furthermore, whereas only 10 μM ATO reduced IL-6 concentrations in the cell culture supernatant (**Figure 1C**), the 5 μM ATO dose strikingly increased the synthesis of IL-10 in ConA-stimulated cells (**Figure 1D**). Then, we investigated whether the changes in both proliferation and cytokine production associated with the highest concentration of ATO were a consequence of immunomodulatory effects of the therapy or a putative induction of cell death. The frequency of dead cells (annexin^+^PI^+^, annexin^-^PI^+^ or annexin^+^PI^-^) treated with both 5 μM and 10 μM ATO was similar to that found in vehicle-treated leukocytes (**Figure 1E**). In addition, the use of 5 μM ATO slightly decreased the cell death of ConA-stimulated cells. Altogether the results showed that ATO modulates the proliferation and synthesis of IL-6 and IL-10 cytokines in stimulated cells without inducing cell death.

### ATO Treatment Attenuates the Clinical Signs of DSS-Induced Colitis

**Figure 2:**
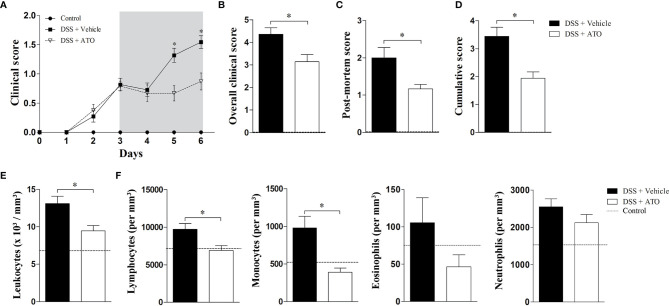
ATO treatment improves the clinical outcome of experimental colitis. C57BL/6 WT mice were exposed to 3% (w/v) DSS in drinking water for 6 days. Mice received oral treatment once daily for three days (days 3-5) with saline (vehicle) or 10 mg/Kg/day ATO. **(A)** Clinical score during the disease course. The hatched area indicates the period of ATO treatment. **(B)** The sum of daily scores was termed as overall clinical score. **(C)** The macroscopic evaluation of colon after euthanasia was termed as post-mortem score. **(D)** The sum of clinical score on day six after DSS administration and post-mortem score was termed as cumulative score. **(E, F)** Total number of circulating white blood cells (leukocytes) **(E)** and differential leukocyte counts **(F)**. The dashed line indicates values from healthy control mice without colitis induction. These results are representative of 3-5 independent experiments with 5 mice per group in each experiment. Unpaired t tests or Mann-Whitney non-parametric t test were used to determine if differences attained significant response. *p < 0.05. ATO, Atorvastatin; DSS, Dextran sodium sulfate.

Since ATO showed anti-inflammatory effects *in vitro*, we next evaluated whether the drug would also have the same effects *in vivo*. For this purpose, WT mice were exposed to 3% DSS in drinking water for 6 consecutive days. After 3 days, when the clinical signs arise, mice were orally administered with vehicle or 10 mg/kg/day of ATO for 3 days, once a day. A dose-response study was conducted previously to select the best dose and route of administration of ATO (data not shown). Treatment with ATO improved clinical signs of colitis when compared to vehicle-treated mice (**Figure 2A**). The overall, post-mortem and accumulated scores confirmed that ATO therapy attenuated the course of colitis (**Figures 2B–D**, respectively).

The number of total circulating white blood cells was reduced in mice that received ATO (**Figure 2E**). A decrease in lymphocytes and monocytes was more significative, followed by a slight decrease of eosinophils (**Figure 2F**).

These findings pointed out that ATO improves the colitis outcome and decreases the number of circulating leukocytes, suggesting a systemic anti-inflammatory role of drug.

### ATO Ameliorates Histopathological Aspects of Colon and Shifts Th17-Polarized Immune Response to Th2 Profile in the Lamina Propria (LP)

**Figure 3:**
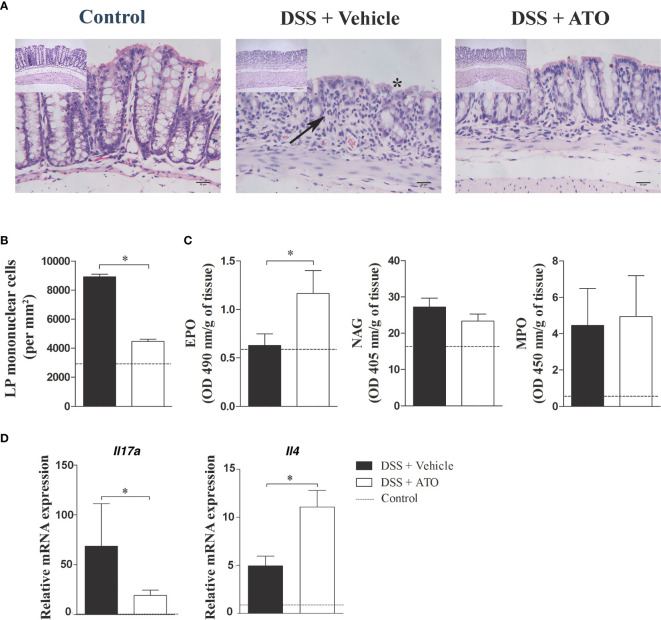
ATO treatment improves the histopathological aspects of the colon, increases the eosinophil activity and IL-4 levels in the gut. C57BL/6 WT mice were exposed to 3% (w/v) DSS in drinking water for 6 days. Mice received oral treatment once daily for three days (days 3-5) with saline (vehicle) or 10 mg/Kg/day ATO. **(A)** Photomicrographs showing the histological analysis of colon. Asterisk indicates areas with absence of crypts. Arrows indicate cell infiltration in the LP. Original magnification: 200x and 400x. **(B)** Number of mononuclear cells in LP per mm^2^ of tissue. **(C)** Activity of EPO, NAG and MPO in colon. The results were expressed by OD (in nm) per gram of tissue. **(D)** Relative mRNA expression of IL-17A and IL-4 in colon. The dashed line indicates the values from healthy control mice without colitis induction. These results are representative of 2 or 3 independent experiments with 5 mice per group in each experiment. Unpaired t test or Mann-Whitney non-parametric t tests were used to determine if differences attained significant response. *p < 0.05. ATO, Atorvastatin; DSS, Dextran sodium sulfate; EPO, Eosinophil peroxidase; LP, Lamina propria; MLN, Mesenteric lymph nodes; MPO, Myeloperoxidase; NAG, N-Acetylglucosaminidase. OD, Optical density.

To verify if the clinical improvements induced by ATO also extended to microscopic anatomy of the colon, we proceed with the histopathological analyzes of the large intestine sections. Healthy control mice had a normal epithelial lining along with parallel and well-structured crypts (**Figure 3A** – left picture). The mean number of LP cells was 2916 ± 123,3 cells/mm^2^ (**Figure 3B**, dashed line). The colon of vehicle-treated DSS-exposed mice showed areas with absence of crypts interspersed with areas of preserved mucosa together with mucin-depleted, irregular and flattened superficial epithelium (**Figure 3A** – central picture). There was also a marked inflammatory infiltrate in the LP of DSS group treated with vehicle with a mean number of 8908 ± 191,8 cells/mm^2^ (**Figure 3B**). However, the treatment with ATO improved mucosal and crypt architecture preservation (**Figure 3A** – right picture), besides decreasing the inflammatory infiltrate in LP with a mean number of 4466 ± 150,6 cells/mm^2^ (**Figure 3B**).

Next, we evaluated whether the cellular composition of the infiltrate was modified following therapy with ATO. Thus, the number of eosinophils, macrophages and neutrophils in the LP was determined using indirect evaluation through EPO, NAG and MPO assays, respectively. As expected, there was an increase in the activity of colonic neutrophils and macrophages in DSS-exposed mice treated with vehicle when compared to healthy controls (**Figure 3C**). Interestingly, the ATO-treated group increased the activity of LP-infiltrating eosinophils, while no changes were detected in macrophages and neutrophils.

Studies regarding experimental models of colitis have described that acute DSS-induced colitis have a predominant Th1/Th17 immune response, while in the chronic phase the inflammation shifts to a Th2 profile (29). Therefore, we investigated whether the different distribution of cells in MLN and spleen was related to a putative cytokine modulation induced by ATO treatment. Interestingly, ATO increased the frequency of CD4^+^IL-17^+^ T lymphocytes in the spleen and MLN, whereas there was a reduction in CD4^+^IFN-γ^+^ T lymphocytes in MLN (**Supplementary Figures 1A, B**). Moreover, we observed a shift in cytokine profile towards a Th2 response after treatment with ATO, by detecting an enhancement of IL-4 at the expense of IL-17 expression in the colon (**Figure 3D**).

Together, the results suggest that ATO improves the morphological aspects of the colon, decreases the number of local inflammatory cell infiltrate, and switch the local responses to a Th2-prone profile, which could be related to immune regulation in the gut during the acute phase of colitis.

### Modification of Immune Cell Subpopulations in Secondary Lymphoid Organs

**Figure 4:**
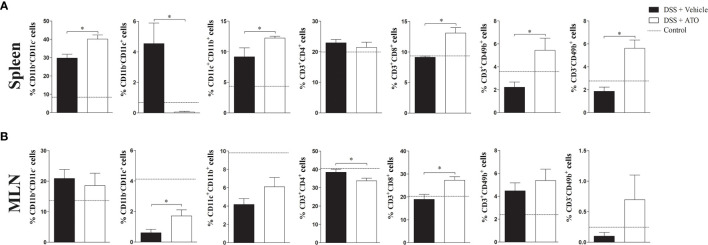
ATO treatment modifies the frequency of several immune cell subpopulations in spleen and MLN. C57BL/6 WT mice were exposed to 3% (w/v) DSS in drinking water for 6 days. Mice received oral treatment once daily for three days (days 3-5) with saline (vehicle) or 10 mg/Kg/day ATO. **(A, B)** The frequency (%) of several immune cell subtypes were assessed by flow cytometry in both spleen **(A)** and MLN **(B)**. Immune cell subsets were determined by expression of surface markers, including: CD11b, CD11c, CD3, CD4, CD8 and CD49b. The dashed line indicates the values from healthy control mice without colitis induction. These results are representative of 3 independent experiments with 5 mice per group. Unpaired t test or Mann-Whitney non-parametric t tests were used to determine if differences attained significant response. *p < 0.05. ATO, Atorvastatin; DSS, Dextran sodium sulfate; MLN, Mesenteric lymph nodes.

Because the number and cell types in the colon were changed after treatment using ATO, we next investigated whether these alterations could be observed in secondary lymphoid organs. The total number of cells in spleen and mesenteric lymph nodes (MLN) was not different between the ATO or vehicle-treated groups. We identified an increased frequency of both CD11b^+^ and CD11b^+^CD11c^+^ monocyte-derived cells, CD8^+^ T lymphocytes, CD3^+^CD49b^+^ NKT cells and CD3^-^CD49b^+^ NK cells in the spleen of ATO-treated mice (**Figure 4A**). In addition, a marked reduction in the frequency of monocyte-derived CD11c^+^ cells were also observed. In turn, ATO therapy decreased the frequency of CD4^+^ T lymphocytes and increased both CD11c^+^ cells and CD8^+^ T lymphocytes in the MLN (**Figure 4B**). These results indicated that ATO has both systemic and local effects on immune cells distribution during the DSS-induced colitis.

### PPAR-α Expression Correlates to Disease Severity

**Figure 5:**
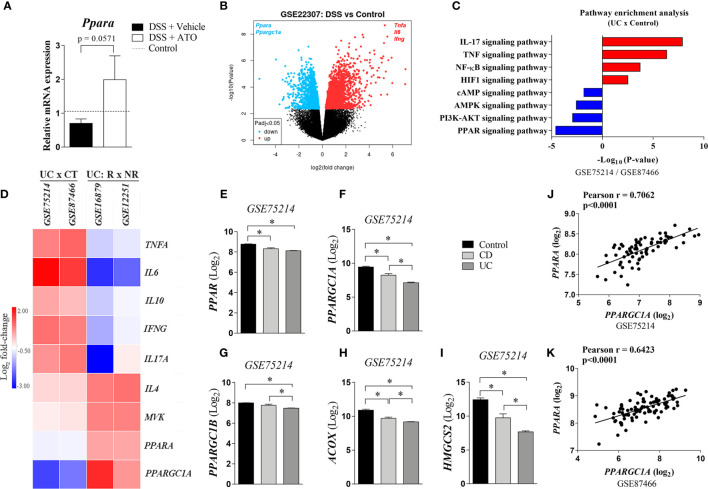
PPAR-α expression is decreased in the inflamed intestine and restored by ATO treatment. **(A)** C57BL/6 WT mice were exposed to 3% (w/v) DSS in drinking water for 6 days. Mice received oral treatment once daily for three days (days 3-5) with saline (vehicle) or 10 mg/Kg/day ATO. Middle colon fragments were used for qRT-PCR analysis and the differential expression of mRNA was defined by the formula 2^-ΔΔCt^. The dashed line indicates the values from healthy control mice without colitis induction. These results are representative of 2 independent experiments with 3-4 mice per group, in each experiment. **(B)** Data were obtained from available dataset under GEO accession GSE22307. Volcano plot shows upregulated (red) and downregulated genes (blue) significantly and differentially expressed at an adjusted p-value cutoff of 0.05. **(C)**. Differentially expressed genes were obtained from GSE75214 and GSE87466 datasets. They were filtered by either logFC<-1 (downregulated genes) or logFC>1 (upregulated genes) and by adjusted p value<0.05. Then, KEGG Pathway enrichment analysis was performed on the up and downregulated genes using the Metascape software. **(D)** Heatmap showing the relative colonic *PPARA* expression between healthy individuals and UC patients (GEO accession GSE75214 and GSE87466), and between responder or non-responder UC patients to infliximab therapy (GEO accession GSE16879 and GSE12251). The expression of inflammatory genes as well as enzymes related to both PPAR signaling and mevalonate pathways in the colon are also depicted. Expression data were preprocessed using a log2 transformation and normalization approach. **(E–I)**. PPARα-related gene expression from colon of healthy, CD and UC patients (GEO accession GSE75214). Expression data were preprocessed using a log2 transformation and normalization approach. **(J, K)**. Correlation analyzes between PPARA and PPARGC1A genes using data from GSE75214 and GSE87466 datasets. Based on Kolmogorov-Smirnov normality test, we selected either Pearson or Spearman analysis for the correlation analyzes. *p<0.05. Unpaired t tests or Mann-Whitney non-parametric t test were used for 2 groups, while ANOVA test was used for 3 or more groups followed by Tukey comparison or Kruskal–Wallis (Dunns) test to determine if differences attained significant response. *ACOX*, Acyl-CoA oxidase 1; CT, control; DSS, Dextran sodium sulfate; *HMGCS2*, 3-Hydroxy-3-Methylglutaryl-CoA Synthase 2; MVK, Mevalonate kinase; NR, Non-responder to Infliximab therapy; PPAR-α/*Ppara*/*PPAR*, Peroxisome proliferator-activated receptor alpha; *Ppargc1a/PPARGC1A*, Peroxisome proliferator-activated receptor gamma coactivator 1-alpha; *PPARGC1B*, Peroxisome proliferator-activated receptor gamma coactivator 1-beta; R, Responder to infliximab therapy; UC, Ulcerative colitis.

Lovett-Racke et al. showed that Th2 cytokine production may be elicited by activating PPAR-α that was sufficient to improve the EAE (30). Then, we investigated if mRNA expression of *Ppara* was modified by ATO treatment. In fact, *Ppara* expression was increased in colon of mice treated with ATO compared to vehicle-treated group (**Figure 5A**). Moreover, our experiments and *in silico* analyzes using normalized GEO datasets (GSE22307) showed that *Ppara* expression was decreased in the colon of DSS-exposed mice (**Figures 5A, B**, respectively). Similar results were found in patients with UC and CD (**Figures 5C, E**) using GEO datasets (GSE75214/GSE87466). Furthermore, while experimental colitis upregulated inflammatory genes in the colon, including *Tnfa*, *Il6* and *Ifng* (**Figure 5B**), UC patients also upregulated *IL17A* and *IL10*, followed by a slight increase in *IL4* and mevalonate kinase (*MVK*) expression (**Figure 5D**). The pathway enrichment analysis confirmed that IL-17 signaling pathway is upregulated in UC patients (**Figure 5C**). Importantly, downregulation of *Ppara* was accompanied by a reciprocal decrease of *Ppargc1a* (**Figure 5B**), a gene that encodes the transcription factor peroxisome proliferator-activated receptor-γ coactivator (PGC)-1α and highly associated with PPAR-α functions (**Figure 5B**). *PPARGC1A* was not only decreased in UC and CD patients but also had a strong correlation with *PPARA* expression (**Figures 5F, J, K**). To support our hypothesis, we also found that other *PPARA*-related genes were downregulated in both UC and CD patients, such as *PPARGC1B*, *ACOX* and *HMGCS2*, which codify PGC-1β, and the enzymes Acyl-CoA oxidase 1 and 3-Hydroxy-3-Methylglutaryl-CoA Synthase 2, respectively (**Figures 5G-I**).

Interestingly, the comparison between UC patients’ responders and non-responders to infliximab therapy, a gold standard drug to treat moderate to severe disease (31), showed that treatment responsiveness was directly associated with the upregulation of *PPARA*, *IL4*, *PPARGC1A* and *MVK* (**Figure 5D**). As expected, infliximab-responsive UC patients downregulated the expression of inflammatory genes in the gut compared to non-responders (**Figure 5D**). Altogether, these results suggest that one of the non-canonical mechanisms mediated by ATO could be the modulation of PPAR-α signaling pathway.

### Genetic Deletion of PPAR-α Abrogates the Beneficial Effects of ATO *In Vitro* and *In Vivo*

**Figure 6:**
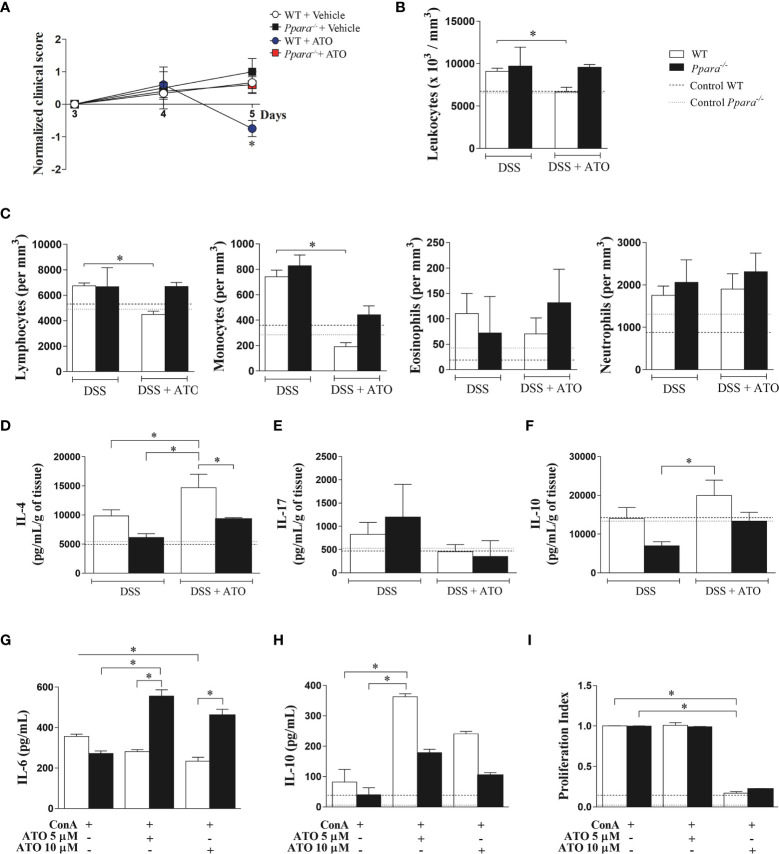
PPAR-α deficiency abrogates the ATO-mediated effects in both experimental colitis and ConA-stimulated splenocytes in *vitro*. **(A)** WT and *Ppara* ^-/-^ mice were exposed to 3% (w/v) DSS in drinking water for 6 days. Mice received oral treatment once daily for three days (days 3-5) with saline (vehicle) or 10mg/Kg/day of ATO. In **(B)** total number of circulating white blood cells (leukocytes) and **(C)** differential leukocyte counts. **(D-F)** Quantification of IL-4 **(D)**, IL-17 **(E)** and IL-10 **(F)** levels in colon tissue lysates. **(G–I)** Splenocytes from C57BL/6 WT and *Ppar^-/-^* naïve mice were stimulated with 3 µg/mL ConA and concomitantly treated with 5 or 10 µM of ATO for 72h. IL-6 **(G)** and IL-10 **(H)** cytokines quantification in the supernatant was performed by ELISA. The proliferation of CFSE-stained cells was evaluated by flow cytometry and expressed as proliferation index **(I)**. The dashed lines indicate values from healthy control WT or *Ppara* ^-/-^ mice without colitis induction or unstimulated cells. These results in A-F are representative of 3 independent experiments with 5 mice per group. The data from graphs G-I are representative of 2 independent experiments that were performed, each one, with splenocytes pooled from 3 WT or *Ppara* ^-/-^ mice. ANOVA test was used followed by Tukey comparison or Kruskal–Wallis (Dunns) test to determine if differences attained significant response. *p < 0.05. ATO, Atorvastatin; ConA, Concanavalin A; DSS, Dextran sodium sulfate; LP, Lamina propria; MLN, Mesenteric lymph nodes; PPAR-α/*Ppara*, Peroxisome proliferator-activated receptor alpha; WT, wild type.

To verify whether PPAR-α mediates the improvement found in ATO-treated group, *Ppara^-/-^* mice were subjected to the same protocol of colitis induction and treatment. The *Ppara* deletion abrogated the amelioration induced by ATO in DSS-exposed mice (**Figure 6A**). Moreover, ATO-treated *Ppara^-/-^* mice did not decrease the number of total circulating leukocytes nor lymphocytes, while the reduction of blood monocytes seemed to be partially dependent on PPAR-α in ATO-treated mice (**Figures 6B, C**). Importantly, *Ppara^-/-^* mice treated with ATO were also unable to increase the IL-4 production in the colon as compared to WT mice treated with ATO, although they slightly decreased IL-17 (**Figures 6D, E**, respectively). In accordance with the *in vitro* results on ATO-induced IL-10 production, ATO-treated WT mice also increased IL-10 levels in the gut following therapy, while *Ppara^-/-^* mice showed a modest cytokine augment (**Figure 6F**).

Finally, ConA-stimulated *Ppara^-/-^* splenocytes did not respond to 5 or 10 μM ATO as seen by the maintenance of increased IL-6 levels and the reduced ability to produce IL-10 after treatment (**Figures 6G, H**, respectively). However, ConA-stimulated *Ppara^-/-^* splenocytes had similar proliferation index compared to WT cells when treated with 5 and 10 μM ATO (**Figure 6I**), indicating that cell proliferation regulated by ATO is independent on PPAR-α functions.

Altogether, our results suggest that the anti-inflammatory role of ATO in colitis seems broadly dependent on the PPAR-α effects and colonic PPAR-α expression is a potential candidate to predict the outcome of IBD treatment.

## Discussion

Current treatments for IBD are focused on the control of inflammatory responses. For this purpose, different strategies and classes of anti-inflammatory or immunosuppressive drugs are used to treat both CD and UC. Consequently, most of patients become either immunosuppressed or unable to restrain secondary infections, besides the occurrence of non-responsiveness to the treatments. This refractoriness to drugs contributes to persistent inflammation, increasing the severity of disease. Thus, more efficient therapies have been investigated thoroughly the last decades. Despite the positive effects of statins in the outcome of IBD are not considered as a recent discovery (16–18, 32, 33), their underlying mechanisms remain poorly understood.

Here, we described that ATO treatment improved several clinical aspects of experimental colitis and promoted a shift from Th17 responses towards to Th2 in the gut of mice exposed to DSS. This shift to Th2 immune response has been described as an important mechanism to ameliorate and prevent several inflammatory conditions. Nath et al. observed that lovastatin treatment induced IL-4 production and GATA3 stabilization in Th2 cells, improving the outcome of EAE (34). Indeed, our experiments and *in silico* analyses revealed, respectively, that both *Il4* expression and IL-4 production at protein level were increased in the colon of ATO-treated mice, while responsiveness to biological therapy was associated with increased *IL4* expression over *IL17*, *IFNG* and other pro-inflammatory genes. Furthermore, the increased activity of eosinophils in colonic LP corroborated the Th2-skewed response after ATO therapy since they are source of IL-4 production in the gut and closely associated to Th2 cells (35).

Lovett-Racke et al. suggested that PPAR-α activation mediates IL-4 synthesis to prevent clinical signs of EAE (30). These results urged us to investigate if PPAR-α was related to the positive effects mediated by ATO therapy. We found that both experimental mice and IBD patients in fact decreased the colonic PPAR-α expression and the full deletion of *Ppara* abrogated the ATO-induced clinical improvements. Importantly, ATO treatment was able to increase the colonic *Ppara* levels in colitis-induced mice, while infliximab-responder UC patients showed an augmented *PPARA* expression compared to non-responder UC patients, suggesting that PPAR-α can be a promising predictive biomarker for clinical responsiveness.

Although we suggest that both infliximab and ATO treatments are associated with PPAR-α upregulation and signaling, these medicines have significantly different pharmacological characteristics and clinical implications. Infliximab is an effective chimeric IgG1 anti-TNF-α monoclonal antibody used to treat moderate to severe IBD patients (31). However, this biological agent has several limitations, including high rates of unresponsiveness and drug discontinuation mainly because of antibodies’ production against infliximab or severe adverse effects (31). ATO, in turn, is a relatively safe drug, orally administrated, economically advantageous, accessible, and less toxic than other drugs (36), increasing the patient adherence. Indeed, our study showed that even with a damaged intestine, ATO was effective in restraining the colitis progression indicating that drug absorption and its consecutive bioavailability were probably not affected. It is also important to highlight that DSS is a chemical model of colitis, but offer several advantages over other experimental methods, including its use to test new treatment options for IBD (37).

Considering the analysis of other genes that could be associated to PPAR-α signaling, mice with experimental colitis, CD and UC patients downregulated *Ppargc1a/PPARGC1A* expression. The product of this gene is PGC-1α, a protein involved with lipid metabolism and mitochondrial biogenesis (38). Infliximab-responder UC patients upregulated *PPARGC1A* expression, suggesting that cell metabolism and, consequently, the cell function can be restored after biological treatment. We also evaluated the expression of other PPAR-α-related genes in both UC and CD patients, including *PPARGC1B, ACOX* (39) and *HMGCS2* (40), all of which were downregulated, indicating that PPAR-α pathway is impaired in IBD, and confirmed by pathway enrichment analysis. The upregulation of MVK in infliximab-responder UC patients, an important enzyme of mevalonate pathway, also indicates that cholesterol synthesis inhibition is not essential for better outcomes of disease, corroborating our hypothesis that ATO plays cholesterol-independent anti-inflammatory roles.

In addition to the immunomodulatory capacity of statins, these drugs also present an immunosuppressive activity, which could be explained by its effects on lowering the number of cells in both gut infiltrate and circulating leukocytes, which were then abrogated in the absence of PPAR-α. In accordance, statins have also been successfully used in cardiac transplantation to reduce the occurrence of organ rejection and hemodynamic disturbances besides the effects of increasing survival rates (41, 42). Additionally, the decreased number of circulating lymphocytes and monocytes could be a consequence of a described action of statins over both LFA-1 and MCP-1, respectively, impairing their homing and recirculation (43, 44). Furthermore, although we did not observe differences in the activity of colonic macrophages (NAG) between vehicle- and ATO-treated mice in our study, we were not able to determine if the increased local levels of IL-4 induced macrophage polarization to a predominant anti-inflammatory M2 phenotype.

Importantly, while several studies have shown that PPAR-α activation is important to decrease the colitis severity in several experimental models (45–48), Qi et al. observed that *Ppar^-/-^* mice as well as those treated with fenofibrate were protected from TNBS- and DSS-induced colitis, and Salmonella Typhi-induced intestinal inflammation (49). We hypothesized that these discrepancies could be a result of different background strains that can cause distinct metabolic response and microbiome composition as previously described (50).

ATO treatment significantly increased the frequency of CD11b^-^CD11c^+^ DCs in the MLN, while increased CD11b^+^CD11c^+^ DCs in the spleen. Studies show that CD11b^-^CD11c^+^ DCs promotes CD8^+^ T cell responses (51), corroborating the increased frequency of CD8^+^ T cells observed in both spleen and MLN of ATO-treated mice. Cytotoxicity induced by CD8^+^ T cells is important to restrain intestinal inflammation during colitis and requires direct interaction with B cells (52). The absence of CD8^+^ T lymphocytes increases intestinal inflammation. Current metabolic approaches also show that PPAR-α is important to preserve CD8^+^ T lymphocytes function (53).

The *in vitro* evaluation showed that ATO plays anti-inflammatory role by two different mechanisms independent on cell death, but dependent on drug concentration, i.e., whereas low dose increases regulatory cytokines such as IL-10, high dose decreases both proliferation and proinflammatory activity in cells. Both IL-6 and IL-10 play an important role in IBD. While the former is increased in IBD and closely related to the differentiation of Th17 cells (54), the lack of IL-10 predisposes to IBD development (55, 56). Although statins have been associated with a decrease in pro-inflammatory cytokines and IL-10 increase (57, 58), no study so far showed their dependency on statin concentration. Moreover, PPAR-α agonists have been described to decrease IL-6 levels in the gut of DSS-exposed mice (45). However, the association between PPAR-α and IL-10 have not been deeply investigated yet, and our study is original by showing this connection not only *in vitro*, but also *in vivo*, thus requiring further investigation. Thus, our experimental results lay at conjunction to *in silico* analysis, indicating that PPAR-α signaling mediates ATO mechanisms that could be explored through clinical managements.

Finally, our study had some limitations regarding the impossibility to perform experiments to demonstrate the direct relationship between ATO and PPAR-α. Even though, previous studies indicate that ATO promotes both upregulation and activation of PPAR-a and that its metabolic and anti-inflammatory effects are mediated by COX-2 enzyme and protein kinase C (PKC) signaling pathway (21, 59, 60). Therefore, the determination of the underlying mechanism of statin-induced modulation of PPAR-a expression and activity, besides the experimental confirmation of the bioinformatic analyses should be performed in future studies of the group.

In conclusion, our results suggest that PPAR-α is essential to the clinical benefits of ATO treatment and it has potential as a predictive biomarker of therapy responsiveness in IBD.

## Data Availability Statement

The datasets presented in this study can be found in online repositories. The names of the repository/repositories and accession number(s) can be found below: https://www.ncbi.nlm.nih.gov/geo/, GSE75214; https://www.ncbi.nlm.nih.gov/geo/, GSE87466; https://www.ncbi.nlm.nih.gov/geo/, GSE16879; https://www.ncbi.nlm.nih.gov/geo/, GSE12251; and https://www.ncbi.nlm.nih.gov/geo/, GSE22307.

## Ethics Statement

The animal study was reviewed and approved by Ethics Committee of the University of São Paulo - campus Ribeirão Preto (protocol number 10.1.499.53.8).

## Author Contributions 

PB designed the project, acquired the data, interpreted and assessed the data, and wrote the manuscript. HS-C provided technical support and performed a critical revision of the manuscript. VN provided significant technical support. MD-S, VA, GB, and CR provided technical support. BG assisted with bioinformatics analysis. JC performed the histological analysis. AN contributed to the project design. CC contributed to the project design, checked the statistical analysis, and edited the manuscript. All authors contributed to the article and approved the submitted version.

## Funding

The research leading to these results has received support from the Conselho Nacional de Desenvolvimento Científico e Tecnológico (CNPq; grant numbers 310174/2016-3, 309583/2019-5, 141050/2013-6, 482390/2013-1, 311882/2013-7) and Fundação de Amparo à Pesquisa do Estado de São Paulo (FAPESP; grant numbers 2010/20162-7, 2013/11042-6, 2017/08651-1 and 2021/08404-0).

## Conflict of Interest

The authors declare that the research was conducted in the absence of any commercial or financial relationships that could be construed as a potential conflict of interest.

## Publisher’s Note

All claims expressed in this article are solely those of the authors and do not necessarily represent those of their affiliated organizations, or those of the publisher, the editors and the reviewers. Any product that may be evaluated in this article, or claim that may be made by its manufacturer, is not guaranteed or endorsed by the publisher.
