# Supplementary material for: Peroxisome Proliferator-Activated Receptor Alpha Mediates the Beneficial Effects of Atorvastatin in Experimental Colitis
Source: Front Immunol. 2021 Aug 9;12:618365. doi: 10.3389/fimmu.2021.618365 (PMC8382038; doi:10.3389/fimmu.2021.618365)
Supplement: Supplementary file 1 [file DataSheet_1.pdf]

Supplementary Material

1 Supplementary Data

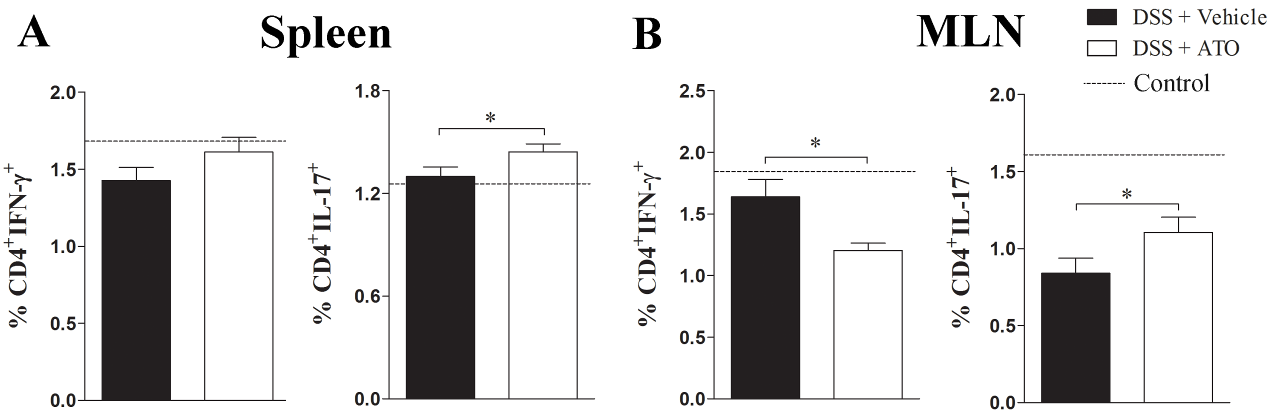

Supplementary figure 1. ATO treatment changes the synthesis of proinflammatory cytokines by CD4^{+} T cells. C57BL/6 WT mice were exposed to 3% (w/v) DSS in drinking water for 6 days. Mice received oral treatment once daily for three days (days 3-5) with saline (vehicle) or 10 mg/Kg/day of ATO. **A-B.** Frequency (%) of IFN-γ- and IL-17-producing CD4^{+} T cells on spleen (A) and MLN (B) after *ex vivo* stimulation with PMA (50 ng/mL) and ionomycin (500 ng/mL) for 4 hours**.** These results are representative of 2 independent experiments with 5 mice per group. Unpaired t test or Mann-Whitney non-parametric t tests were used to determine if differences attained significant response. *$p<0.05$. **ATO:** Atorvastatin; **DSS:** Dextran sodium sulfate; **MLN:** Mesenteric lymph nodes.
